# Observer Influence with Other Variables on the Accuracy of Ultrasound Estimation of Fetal Weight at Term

**DOI:** 10.3390/medicina57030216

**Published:** 2021-02-27

**Authors:** Mariola Sánchez-Fernández, Maria E. Corral, Longinos Aceituno, Marina Mazheika, Nicolás Mendoza, Juan Mozas-Moreno

**Affiliations:** 1 Obstetrics and Gynecology Service, La Inmaculada Hospital, Huércal-Overa, 04600 Almería, Spain; mariola333@hotmail.com (M.S.-F.); ecesegade@gmail.com (M.E.C.); laceitunov@sego.es (L.A.); 2 Department of Obstetrics and Gynecology, University of Granada, 18016 Granada, Spain; marinamazheika@ugr.es (M.M.); nicomendoza@ugr.es (N.M.); 3 Obstetrics and Gynecology Service, Virgen de las Nieves University Hospital, 18014 Granada, Spain; 4 Consortium for Biomedical Research in Epidemiology & Public Health (CIBER Epidemiology & Public Health-CIBERESP), 28029 Madrid, Spain; 5 Biohealth Research Institute (Ibs.GRANADA), 18014 Granada, Spain

**Keywords:** birth weight, fetal weight, ultrasound fetal biometry, estimated fetal weight, fetal macrosomia, fetal microsomia, fetal growth restriction

## Abstract

*Background and Objectives:* The accuracy with which the estimation of fetal weight (EFW) at term is determined is useful in order to address obstetric complications, since it is a parameter that represents an important prognostic factor for perinatal and maternal morbidity and mortality. The aim of this study was to determine the role of the experienced observers with other variables that could influence the accuracy of the ultrasound used to calculate EFW at term, carried out within a period of seven days prior to delivery, in order to assess interobserver variability. *Materials and Methods:* A cross-sectional study was performed including 1144 pregnancies at term. The validity of the ultrasound used to calculate EFW at term was analyzed using simple error, absolute error, percentage error and absolute percentage error, as well as the percentage of predictions with an error less than 10 and 15% in relation to maternal, obstetric and ultrasound variables. *Results:* Valid predictions with an error less than 10 and 15% were 74.7 and 89.7% respectively, with such precision decreasing according to the observer as well as in extreme fetal weights. The remaining variables were not significant in ultrasound EFW at term. The simple error, absolute error, percentage error and absolute percentage error were greater in cases of extreme fetal weights, with a tendency to overestimate the low weights and underestimate the high weights. *Conclusions:* The accuracy of EFW with ultrasound carried out within seven days prior to birth is not affected by maternal or obstetric variables, or by the time interval between the ultrasound and delivery. However, accuracy was reduced by the observers and in extreme fetal weights.

## 1. Introduction

Obstetric ultrasound is considered a routine test to evaluate fetal morphology, gestational age, fetal growth, and estimated fetal weight (EFW) throughout pregnancy. The accuracy with which EFW at term is determined is useful in order to address obstetric complications, since it is a parameter that represents an important prognostic factor for perinatal and maternal morbidity and mortality. Macrosomic fetuses are at greater risk of suffering from shoulder dystocia during delivery and the associated morbidities such as brachial palsy, facial paralysis, neurological alterations, and bone fractures. Moreover, maternal complications associated with fetal macrosomia include a higher rate of cesarean section, instrumental deliveries, uterine atony, postpartum infections, traumas of the birth canal and severe perineal tears [1,2]. In the opposite case, a fetal weight below 2500 g (low birth weight) and intrauterine growth restriction (IUGR) could lead to neonatal complications such as respiratory distress, hypoglycemia, respiratory infection, or the need for assisted ventilation [3].

Other aspects of the birth that are determined by the EFW include the type of delivery (elective cesarean section, induced delivery, or spontaneous birth), as well as the chosen obstetric approach when there is a fetal biometry with a low EFW, which may vary from the induction of labor due to IUGR, attempts to delay delivery in order to allow the fetus to mature, or the transfer of the pregnant woman to a specialized center with a neonatal intensive care unit. Therefore, adequate accuracy in the calculation of EFW could serve to reduce maternal and perinatal morbidity and mortality associated with extreme fetal weights [1,2,3].

EFW is calculated through fetal biometry using ultrasound, and there are multiple formulas for its estimation. For instance, Hadlock et al. [4] developed several formulas to EFW, which included measures of the biparietal diameter (BPD), head circumference (HC), abdominal circumference (AC) and femur length (FL). The generally accepted approach is to combine the ultrasonographic measures of several parameters, as well as to use logarithmic formulas to reduce error in the estimation. However, some maternal, obstetric and ultrasound performance variables have been considered as a potential influence of the accuracy of EFW. Among them, the following have been considered: ethnic differences [5,6]; maternal obesity [7,8,9,10]; cases of extreme fetal weights [11,12,13]; mount of amniotic fluid [11,12,13,14]; sex of the fetus [7,15,16,17]; fetal presentation [18,19]; thickness and location of the placenta [12]; and time interval between ultrasound and delivery [11]. However, when evaluating the role of the observers, it is always done by comparing among technicians, residents and staff physicians [7,12,20,21].

The aim of this study was to determine the role of the experienced observers with other variables that could influence the accuracy of a two-dimensional ultrasound used to calculate EFW at term, carried out within a period of seven days prior to delivery, in order to assess interobserver variability.

## 2. Materials and Methods

### 2.1. Study Design and Patients

In this cross-sectional study, a review was conducted of the all the most recent clinical histories of 1650 pregnant women who delivered during the period 2017–2018, at the public hospital La Inmaculada, Huércal-Overa, Almería, Spain, where 1300 births are attended annually, and do not carry out Obstetrics teaching activity. The sample was selected from a target population of pregnant women who met the inclusion criteria and did not present any exclusion criteria. The inclusion criteria were full-term delivery (37–42 weeks of gestation); single fetus; intact amniotic sac; and fetal biometry performed during the prenatal consultation. In this biometry the BPD, AC, and FL were included, which calculated the EFW within a maximum time period of seven days between the assessment and delivery. The exclusion criteria were preterm delivery (<37 weeks of gestation); post-term delivery (>42 weeks of gestation); multiple pregnancy; rupture of the amniotic sac before ultrasound; fetal or uterine malformation; fetal death; acromion presentation or an elapsed time of more than seven days between the ultrasound scan and delivery. The final sample that met all the inclusion criteria consisted of 1144 pregnant women.

The information collected to create the database with which the analysis was conducted included maternal, obstetric, and ultrasound variables. The maternal variables considered were age; ethnicity; height; weight; body mass index (BMI); and parity. The obstetric variables were fetal sex; fetal presentation; placental location; quantity of amniotic fluid; EFW; and birth weight (BW). The ultrasound variables were the number of days that had elapsed between ultrasound and delivery, and the observer who performed the ultrasound.

The EFW and the BW were classified—independently of gestational age—as low BW or microsomic (<2500 g), macrosomic (>4000 g), and normal BW (2500–4000 g). According to the percentile distribution by sex, single pregnancy, and gestational age, and using national BW tables as a reference, the neonate was classified as small for gestational age (SGA) when the percentile was <10, large for gestational age (LGA) when the percentile was >90 and adequate for gestational age (AGA) when the percentile was between 10 and 90.

### 2.2. Instruments

All examinations were carried out with the same real-time ultrasound model (Toshiba Medical System Xario SSA-660A; Otawara, Tochigi, Japan) with a 3.5 MHz transducer. The formula used to calculate the EFW was that proposed by Hadlock et al. [4] (Hadlock 2): Log10 EW = 1.335 − (0.0034 × AC × FL) + (0.0316 × BPD) + (0.0457 × AC) + (0.1623 × FL). A single observer calculated each EFW. The observers were eight staff gynecologists with over six years of experience in fetal biometrics and their consultations were scheduled on a rotational basis. The midwife determined the BW for each delivery using the same scale, which was repeatedly calibrated and located in the delivery room area.

Gestational age was calculated according to the last menstrual period and was corrected when there was a discrepancy of more than seven days between this and the date established by the first-trimester ultrasound (based on the cranio-caudal length). The location of the placenta was classified as anterior when the insertion of the placenta was anterior or fundal, whereas it was considered posterior when the insertion of the placenta was totally posterior. The quantity of amniotic fluid was estimated according to the four quadrants technique developed by Phelan et al. [22] in which it was classified as normal when the amniotic fluid index (AFI) was between 5 and 21 cm, scarce when it was <5 cm, and abundant when it was >21 cm. The EFW at term were routinely performed in the hospital following the local protocol.

To measure the BPD, a median transaxial plane was taken at the point where the midline was interrupted by the septum pellucidum and the thalami. The AC was carried out in the plane that passes at the level of the liver, looking at the fetal portal system and with perpendicular cut of the rachis. This circumference was estimated indirectly, that is, with the antero- posterior and transverse diameters of the abdomen. The calipers were placed on the outer table of the parietals for the BPD and on the fetal skin for the AC. FL was measured along the major axis of the diaphysis, avoiding curvature from the greater trochanter to the lateral condyle, and avoiding the head of the femur and the distal epiphysis.

Maternal BMI was calculated using the maternal weight and height measurements obtained at the first antenatal appointment, according to the formula: BMI = weight (kg)/height^2^ (m^2^). BMI categories were defined as follows: normal weight (BMI 18.5–24.9); overweight (BMI 25–29.9), obese (BMI > 29.9), and underweight (BMI < 18.5).

### 2.3. Statistical Analysis

A descriptive analysis was conducted for each variable with all of the data, using absolute and relative frequency distributions for the categorical variables and the number of cases, means, standard deviations, and ranges for the quantitative variables. The assumption of normality of the fetal weight variable was made through the Kolmogorov–Smirnov test. The Student t test for paired samples was used in order to compare the EFW and BW means. The one-way ANOVA test was performed to compare variables that presented more than two categories, considering only multiple comparisons when the ANOVA test was significant. Pearson’s correlation coefficient was used to study the correlation between the calculation of the ultrasound EFW and BW. To identify the variables that could be associated with the ultrasound EFW within 10% of BW, a multiple logistic regression analysis was performed, selecting the independent variables according to statistical and epidemiological criteria. The OR of the crude and adjusted models with its corresponding CI (95%) were presented.

In order to obtain validity measures that take into account all the estimates, calculations were made of the average of: simple error (SE = EFW − BW/n), absolute error (AE) (which includes that difference in absolute value), percentage error (PE = (EFW − BW/BW) × 100), and absolute percentage error (APE) (which reflects this percentage in absolute value), in the total sample, in cephalic and breech fetal presentations, and in the extreme fetal weights. The percentages of estimates that had an error less than 10 and 15% (estimates that fell within the intervals {0.90 × BW, 1.10 × BW} and {0.85 × BW, 1.15 × BW}) were also calculated. The X2 test or Fisher´s exact test were used to compare the proportions of EFW within 10 and 15% of the BW according to the different variables (maternal, obstetric, classification of fetal weight and ultrasound) considered. The level of significance for all the analyses was set at *p* < 0.05. When differences were established between the observers, Observer 1 was taken as a reference for obtaining the highest percentage of EFW within 10% of the BW, and the data were adjusted for multiple comparisons (using the Bonferroni correction, level of significance *p* < 0.01). The BW was used as a reference to confirm the validity of EFW. Data analyses were conducted using the Statistical Program SPSS version 20.0 (IBM, New York, NY, USA).

### 2.4. Ethics

The study was conducted in accordance with the Declaration of Helsinki, and the protocol (ECOGRAFIABIDIMENSIONAL16) was approved on October 11, 2016 by the reference Research Ethics Committee.

## 3. Results

The mean maternal age was 29.5 (±5.8) years and the mean weight, height, and BMI were 67.8 (±12.9) kg, 1.64 (±0.04) m and 25.5 (±4.7) kg/m^2^, respectively. Overall, the mean gestational age at delivery was 280 (±8.5) days. The average interval between ultrasound examination and delivery was 39.2 (±1.1) and 39.6 (±0.9) weeks, respectively. The mean number of days that had elapsed from the time of the ultrasound until birth was 3.1 (±2.1) days. The BW ranged from 1800 to 5120 g with a normal distribution and a mean of 3386.5 (±462.6) g. The mean EFW was 3371.6 (±408.3) g, and no significant differences were found between the means of EFW and BW. For the whole sample, Pearson’s correlation coefficient between the ultrasound EFW calculated and BW was *r* = 0.747, which indicates a linear, high and positive association (*p* < 0.001).

The mean AE of the ultrasound EFW was 235.4 g, and it was found that 40.8% of estimates had an error higher than the mean error. In comparison with BW, 9.5% of ultrasound EFW had an error greater than ±500 g. Table 1 shows the BW, EFW, SE, AE, PE, and APE, both in the whole sample and subdivided according to fetal presentation. It was observed that in breech presentations, EFW had higher error than in cephalic presentations, although this difference failed to reach significance.

When the ANOVA test was performed, it was only statistically significant for the variables fetal weight, fetal weight percentiles and observer. In the case of low or excessive fetal weights (low birth weight, macrosomic, SGA, and LGA categories) there were differences between EFW and BW (*p* < 0.01), whilst there was also a higher incidence of different types of errors, showing a tendency towards the general overestimation of EFW in the lower weight range and an underestimation at the high weights range (Table 2).

Regarding the validity of the predictions according to the maternal variables (ethnicity, parity, and BMI), it was observed that the differences for an error lower than 10 and 15% were not significant, with the exception of multiparous pregnant women only when taking into account an EFW within 10% of the BW (*p* < 0.05) (Table 3).

When the obstetric variables (fetal sex, fetal presentation, location of the placenta, and quantity of amniotic fluid) were considered, no differences were found for the percentage of valid predictions of EFW within 10 and 15% of the BW (Table 4).

In the whole sample, 74.7% of EFW calculations were within 10% of the BW, which increased to 89.7% for EFW that fell within 15% of the BW. Similar percentages were found for both normal BW and AGA, but these percentages decreased in the case of extreme BW. When their frequencies were compared, it was found that these differed significantly for cases of low BW, macrosomia, SGA, and LGA respectively, when considering valid calculations of EFW that fell within 10 and 15% of the BW (Table 5).

In terms of the days that had elapsed between ultrasound and birth, for the seven-day interval, there was no difference in valid estimates for either the percentage of EFW within 10 or 15% of the BW. Concerning the validity of predictions according to the observer, the percentage of EFW within 10% of the BW was higher for Observer 1 (83.5%) than the other observers. Therefore, Observer 1 was taken as the reference for comparisons with the other observers. For Observers 4, 5, 6, 7 and 8, significant differences (*p* < 0.05) were found when the lower error limit of 10% was taken into account when comparing them with Observer 1 taken as reference. Regarding the percentage of valid predictions with an error less than 15%, significant differences (*p* < 0.05) were found between Observers 5 and 7 versus Observer 1 taken as reference. When the adjustment was made for multiple comparisons between observers (Bonferroni test with level of significance *p* < 0.01), Observer 1 showed statistically significant differences between the EFW and the BW with an error lower than 10% with respect to Observers 4, 7 and 8. However, when also making this correction for the multiple comparisons, no significant differences were obtained when taking into account cases in which the error difference between EFW and BW was within 15% (Table 6).

**Figure 1:**
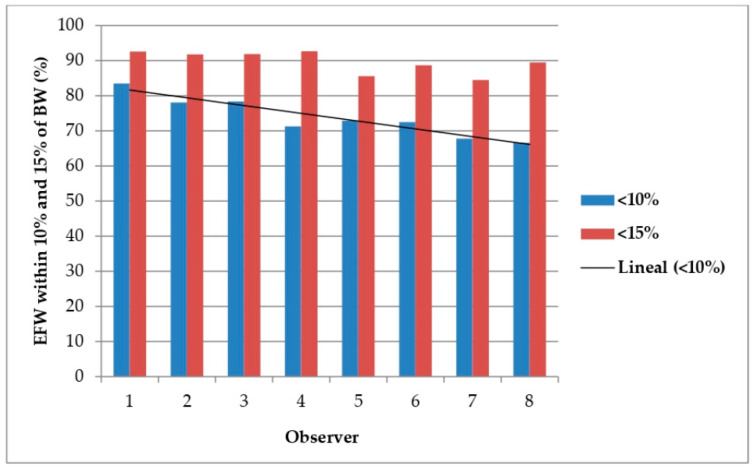
Valid predictions of EFW within 10% and 15% of BW (%) according to observer.

Figure 1 shows valid predictions of EFW within 10 and 15% of BW according to observer.

**Figure 2:**
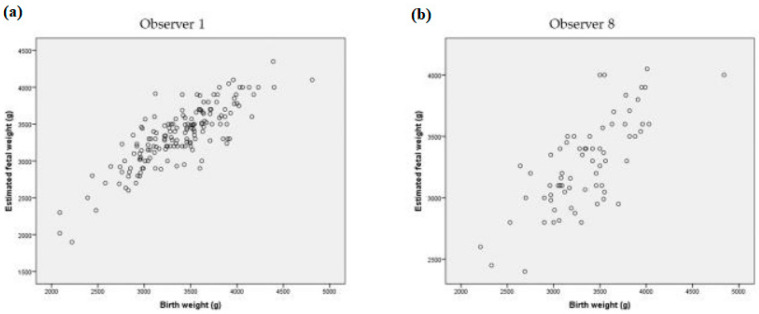
Comparison of correlation between EFW (g) and BW (g) by Observer 1 (**a**) taken as a reference and Observer 8 (**b**).

Figure 2 shows the comparison of the correlation that was reached between EFW and BW by Observer 1 taken as a reference (*r* = 0.828) and Observer 8 (*r* = 0.716) that had the lowest rate of valid predictions of EFW within 10% of BW.

Table 7 shows the crude and adjusted models for ultrasound EFW within 10% of BW. After adjusting the regression model, X2 *p* < 0.001 was obtained in the global hypothesis contrast, so the variability in the valid predictions of EFW within 10% of BW was due to the relationship with the fetal weight by percentile, the range of fetal weight and the observer, which were the variables selected in the model.

## 4. Discussion

Ultrasound is a valuable tool for prenatal care, as it plays an important role not only in assessing fetal growth (by calculating the EFW), but also in the detection of potential obstetric pathologies. The EFW, using two-dimensional ultrasound, is suitable for planning the management of pregnancy and mode of delivery, since it is a crucial variable affecting perinatal morbidity and mortality, particularly in large/small fetuses.

Although the Hadlock’s method to EFW is superior to the more modern methods for predicting the BW percentile [23], in recent years a wide variety of mathematical equations have been developed to improve the accuracy of EFW by using various fetal parameters, however, none of these methods have been proven to be superior to the others [24]. The EFW is assumed to be accurate as long as the percentage of valid predictions is around 65% (considering an error of less than 10%). It should be taken into account that the effectiveness could vary according to the different variables considered [20]. On the other hand, in a study carried out by Kehl et al. [24] that compared the EFW and the BW, it was proposed that a valid prediction rate of 80% is an acceptable limit considering an error of less than 10% of the BW, while a maximum limit of only 5% was established for errors greater than ±500 g. Our results are between these ranges, reiterating a high degree of error in EFW by ultrasound at term. The EFW accuracy was reduced by observer and in cases of extreme fetal weight. Nevertheless, found no significant differences in terms of the other variables analyzed such as maternal ethnicity, BMI, fetal sex, fetal presentation, location of the placenta, amount of amniotic fluid, or the number of days that had elapsed within the seven-day interval between the ultrasound examination and birth. Conversely, with regard to parity, in our study, differences were found, but only for an error lower than 10% of the BW, which could be explained by the fact that ultrasonographers have a tendency to be more meticulous when performing ultrasound in multiparous women, since these women are older and have higher risk of hypertension, gestational diabetes, macrosomic fetuses, shoulder dystocia, and severe perineal tears.

There is conflicting evidence regarding the influence of sonographer’s experience on EFW [7]. Although there are studies that conclude that this variable does not influence EFW [12,25], most do find a difference in favor of staff physicians [12,20,21]. Our analyses revealed significant differences in the accuracy of EFW among the different experienced observers. The use of untrained observers to EFW without evaluation is inappropriate, however, it is difficult to determine how long a sonographer can be considered as experienced for EFW, but it must be noted that all observers in our study had over six years of experience in fetal biometrics. The differences found could be a result the time spent conducting the ultrasound, which could in turn be due to the widespread work overload experienced by consultants in the prenatal clinic. It has been proven that a poor image quality has an impact on the accuracy of fetal measurements and it is likely to be a factor in interobserver variability. Likewise, one possibility to try to improve the quality of fetal measurements is to perform an audit on a sequential sample following widely accepted quality criteria, especially head and AC measurements. In this sense, it has been found that the sonographers, after receiving comments on the number of satisfactory measurements and on the quality criteria not met, improved the proportion of images sent that met all the quality criteria. Said study established that there was considerable variability in the quality of measurement between centers and that performance could be improved [26].

The interest in calculating the EFW at term is fundamentally to diagnose extreme fetal weights, which can compromise the development of labor, as well as perinatal and maternal morbidity and mortality. However, the EFW results at term are not entirely accurate, with a tendency to overestimate the low weights and underestimate the high weights, as other authors have also shown [27]. Clinicians should be aware of this and advise patients of the necessary precautions, which especially affect extreme fetal weights, because they are precisely where the precision of the EFW is most important in its management. Taking our results into account, in case of extreme fetal weights detected, clinicians should make several measurements of each ultrasound parameter for calculating EFW and average the result.

One limitation of this study concerns the fact that there was no random allocation of the patients to the observers who conducted the ultrasound. Nonetheless, the data were collected under conditions of routine clinical practice, which means that different professionals attended the consultations on a rotational basis, without taking into account the pathology. Moreover, they were blind to the fact that they were going to be subsequently evaluated, and the study was conducted with all the population of pregnant women.

The precise variables that may influence EFW still remain unclear, and some authors have even argued that the diagnostic validity of ultrasound for EFW at term has already reached the highest possible level of accuracy [24], thus leaving no potential for further improvements. On the other hand, two-dimensional ultrasound, although it has limitations, is still the most widely used method for EFW at term, as it is relatively cost-effective and easily accessible.

## 5. Conclusions

The results of this study indicate that the accuracy for EFW by ultrasound performed within seven days before birth is not compromised by maternal factors, obstetric variables, or the number of elapsed days in the seven-day interval between ultrasound and delivery. However, the observers and extreme fetal weights (low birth weight/SGA, or macrosomia/LGA) are both factors that decrease the accuracy of this ultrasound technique. Therefore, efforts should be made to minimize the effects of these variables in order to improve the performance of EFW.

## Figures and Tables

**Table 1:** Birth weight, estimated fetal weight and types of errors in the total sample and according to fetal presentation.

Variable	Total Sample (*N* = 1144)	Cephalic (*n* = 1112)	Breech (*n* = 32)
BW (g) Mean (SD)	3386.5 (462.6)	3390.8 (461.5)	3237.2 (483.9)
EFW (g) Mean (SD)	3371.6 (408.3)	3377.6 (406.3)	3162.8 (425.9)
SE (g) Mean (SD)	−16.72 (302.5)	−13.9 (302.3)	−111.9 (300.1)
AE (g) Mean (SD)	235.4 (190.4)	234.5 (190.9)	265.7 (173.4)
PE (%) Mean (SD)	−0.1 (11.5)	−0.1 (11.6)	−2.9 (9.2)
APE (%) (SD)	7.2 (9.1)	7.2 (9.2)	8.1 (5.1)

Abbreviations: BW, birth weight; SD, standard deviation; EFW, estimated fetal weight; SE, simple error; AE, absolute error; PE, percentage error; APE, absolute percentage error.

**Table 2:** Birth weight, estimated fetal weight and types of errors in extreme fetal weights.

Variable	Low Birth Weight (*n* = 34)	Macrosomic (*n* = 100)	SGA (*n* = 88)	LGA (*n* = 161)
BW (g) Mean (SD)	2315.3 (15.2)	4246.4 (228.5)	2550.0 (229.9)	4105.4 (262.7)
EFW (g) Mean (SD)	2521.4 (282.7) ^a^	3905.8 (291.1) ^a^	2773.4 (318.7) ^a^	3826.0 (303.2)^a^
SE (g) (SD)	176.7 (261.4)	−340.7 (339.6)	212.1 (232.8)	−279.1 (319.6)
AE (g) (SD)	260.5 (174.9)	384.2 (288.9)	202.1 (173.7)	332.8 (262.7)
PE (%) (SD)	7.6 (11.3)	−7.5 (7.9)	8.3 (9.4)	−6.4 (7.6)
APE (%) (SD)	11.3 (7.4)	8.9 (6.4)	10.4 (6.9)	7.9 (5.9)

Abbreviations: SGA, small for gestational age; LGA, large for gestational age; BW, birth weight; SD, standard deviation; EFW, estimated fetal weight; SE, simple error; AE, absolute error; PE, percentage error; APE, absolute percentage error; ^a^ *p* < 0.01 in all cases between EFW and BW.

**Table 3:** Valid predictions of fetal weight according to maternal variables.

Variable	Category	Total *N* (%)	EFW within 10% of BW *n* (%)	*p*	EFW within 15% of BW *n* (%)	*p*
Ethnicity	Caucasian	802 (70.1)	598 (74.5)	-	723 (90.2)	-
	Arab	130 (11.4)	96 (73.8)	0.824	113 (86.9)	0.249
	South American	122 (10.7)	95 (77.8)	0.432	110 (90.2)	0.996
	Gypsy	77 (6.7)	57 (74.1)	0.918	68 (88.3)	0.608
	Afro American	13 (1.1)	8 (61.5)	0.366	12 (92.3)	1.0
Parity	Nulliparous	516 (45.1)	367 (71.2)	-	453 (87.8)	-
	Multiparous	628 (54.9)	487 (77.5)	0.013 ^a^	573 (91.2)	0.056
BMI	Normal	630 (55.0)	467 (74.1)	-	566 (89.8)	-
	Overweight	335 (29.3)	244 (72.8)	0.773	303 (90.4)	0.682
	Obesity	179 (15.7)	143 (79.9)	0.419	157 (87.7)	0.291
	Underweight	14 (1.2)	13 (92.8)	0.130	14 (100.0)	0.381

Abbreviations: EFW, estimated fetal weigh; BW, birth weight; BMI, body mass index. ^a^ *p* < 0.05 in EFW within 10% of BW between nulliparous and multiparous pregnant women.

**Table 4:** Valid predictions of fetal weight according to obstetric variables.

Variable	Category	Total *N* (%)	EFW within 10% of BW *n* (%)	*p*	EFW within 15% of BW *n* (%)	*p*
Fetal Sex	Male	585 (51.1)	440 (75.2)	-	533 (91.1)	-
	Female	559 (48.9)	414 (74.1)	0.654	493 (88.2)	0.105
Fetal P	Cephalic	1112 (97.2)	834 (75.0)	-	997 (89.6)	-
	Breech	32 (2.8)	20 (62.5)	0.109	29 (90.6)	0.859
Placental L	Anterior	684 (59.8)	520 (76.1)	-	619 (90.5)	-
	Posterior	460 (40.2)	334 (72.6)	0.193	407 (88.5)	0.271
AFI	Normal	1059 (92.6)	789 (74.5)	-	953 (89.9)	-
	Scarce	34 (3.0)	24 (70.6)	0.607	28 (82.3)	0.148
	Abundant	51 (4.5)	41 (80.4)	0.344	45 (88.2)	0.684

Abbreviations: EFW, estimated fetal weight; BW, birth weight; fetal P, fetal presentation; placental L, placental location; AFI, amniotic fluid index.

**Table 5:** Valid predictions of fetal weight according to the classification of fetal weight.

Variable	Category	Total *N* (%)	EFW within 10% of BW *n* (%)	*p*	EFW within 15% of BW *n* (%)	*p*
Fetal weight	Normal	1010 (88.3)	778 (77.1)	-	920 (91.1)	-
	Low	34 (3.0)	14 (41.2)	<0.01 ^a^	23 (67.6)	<0.01 ^a^
	Macrosomic	100 (8.7)	62 (62.0)	<0.01 ^a^	83 (83.0)	<0.01 ^a^
FW percentiles	AGA	895 (78.2)	700 (78.2)	-	823 (91.9)	-
	SGA	88 (7.7)	47 (53.4)	<0.01 ^a^	63 (71.6)	<0.01 ^a^
	LGA	161 (14.1)	107 (66.4)	<0.01 ^a^	140 (86.9)	<0.05 ^b^

Abbreviations: EFW, estimated fetal weight; BW, birth weight; FW percentiles, fetal weight according to percentiles; AGA, adequate for gestational age; SGA, small for gestational age; LGA, large for gestational age. ^a^ *p* < 0.01; ^b^ *p* < 0.05.

**Table 6:** Valid predictions of fetal weight according to ultrasound variables.

Variable	Category	Total *N* (%)	EFW within 10% of BW *n* (%)	*p*	EFW within 15% of BW *n* (%)	*p*
Observer	1	162 (14.2)	136 (83.5)	-	150 (92.6)	-
	2	73 (6.4)	57 (78.1)	0.277	67 (91.8)	0.829
	3	185 (16.2)	145 (78.4)	0.187	170 (91.9)	0.808
	4	164 (14.3)	117 (71.3)	0.006 ^a,b^	152 (92.7)	0.975
	5	229 (20.0)	167 (72.9)	0.010 ^a^	196 (85.6)	0.033 ^a^
	6	178 (15.6)	129 (72.5)	0,011 ^a^	158 (88.7)	0.227
	7	84 (7.3)	57 (67.8)	0.004 ^a,b^	71 (84.5)	0.047 ^a^
	8	69 (6.0)	46 (66.6)	0.003 ^a,b^	62 (89.5)	0.488
Days elapsed	0	91 (8.0)	66 (72.5)	-	79 (86.8)	-
	1	213 (18.6)	163 (76.5)	0.459	195 (91.5)	0.205
	2	214 (18.7)	167 (78.1)	0.300	195 (91.1)	0.255
	3	192 (16.8)	144 (75.0)	0.657	171 (89.1)	0.582
	4	148 (12.9)	103 (69.6)	0.629	132 (89.2)	0.579
	5	93 (8.1)	75 (80.6)	0.193	87 (93.5)	0.124
	6	97 (8.5)	73 (75.2)	0.670	85 (87.6)	0.867
	7	96 (8.4)	63 (65.6)	0.308	82 (85.4)	0.783

Abbreviations: EFW, estimated fetal weight; BW, birth weight; ^a^ *p* < 0.05; ^b^ percentage difference (statistically significant) between observers for EFW within 10% of BW adjusted according to Bonferroni correction (*p* < 0.01).

**Table 7:** Crude and adjusted models for ultrasound EFW within 10% of BW.

Variable	Model		Crude			Adjusted		
	Reference Category	Risk Category	OR	Crude Model CI (95%)	*P*	OR	Adjusted Model CI (95%)	*p*
Fetal weight	Normal	Low	0.487	0.317–0.748	<0.001 ^a^	2.029	0.910–5.260	0.043 ^c^
		Macrosomic	2.331	1.054–5.153	0.037 ^c^	1.520	0.511–4.532	0.450
FW percentiles	AGA	SGA	0.552	0.384–0.794	0.01 ^c^	0.756	0.429–1.332	0.333 ^c^
		LGA	1.729	1.016–2.941	0.04 ^c^	1.698	0.791–3.645	0.175
Observer	1	2	0.382	0.199–0.735	0.004 ^b^	0.371	0.191–0.722	0.004 ^b^
		3	0.561	0.266–1.185	0.130	0.556	0.260–1.190	0.130
		4	0.552	0.300–1.116	0.046 ^a^	0.557	0.310–1.075	0.083
		5	0.803	0.439–1.470	0.478	0.743	0.401–1.380	0.347
		6	0.743	0.416–1.325	0.314	0.719	0.398–1.299	0.274
		7	0.760	0.417–1.385	0.368	0.784	0.426–1.445	0.436
		8	0.947	0.481–1.867	0.876	0.883	0.441–1.766	0.724

Abbreviations: EFW, estimated fetal weight; BW, birth weight; OR, odds ratio; CI, confidence interval; FW percentiles, fetal weight according to percentiles; AGA, adequate for gestational age; SGA, small for gestational age; LGA, large for gestational age; ^a^ *p* < 0.001; ^b^ *p* < 0.01; ^c^ *p* < 0.05.

## Data Availability

No new data were created or analyzed in this study. Data sharing is not applicable to this article.
